# Artificial intelligence-driven detection of microplastics in food: A comprehensive review of sources, health risks, detection techniques, and emerging artificial intelligence solutions

**DOI:** 10.1016/j.fochx.2025.102687

**Published:** 2025-06-24

**Authors:** Himani Rawat, Ashish Gaur, Narpinder Singh, Manickam Selvaraj, Arun Karnwal, Gaurav Pant, Tabarak Malik

**Affiliations:** a Department of Microbiology, Graphic Era (Deemed to be University), Dehradun, India; b Department of Biotechnology, Graphic Era (Deemed to be University), Dehradun, India; c Department of Food Science and Technology, Graphic Era (Deemed to be University), Dehradun, India; d Department of Chemistry, Faculty of Science, King Khalid University, Abha 61413, Saudi Arabia; e Research Centre for Advanced Materials Science (RCAMS), King Khalid University, AlQura'a, P.O. Box 960, Abha, Saudi Arabia; f Department of Biomedical Sciences, Institute of Health, Jimma University, Ethiopia; g Division of Research and Development, Lovely Professional University, Phagwara, Punjab, 144401 India

**Keywords:** Artificial intelligence, Food safety, Health effects, Microplastics

## Abstract

Microplastic contamination in food is an escalating concern due to associated environmental and health risks, with a rising global plastic production projected to exceed 2.1 billion tons annually by 2060. This makes it essential to have effective detection and identification of microplastics for determining environmental risk and secure food safety. This study is an effort to compare conventional methods (optical detection, thermo-analytical, hyperspectral imaging) with advanced techniques (Fourier transform infrared spectroscopy, pyrolysis-gas chromatography–mass spectrometry, Raman spectroscopy) in the detection of microplastics in food. While conventional methods are effective enough in providing qualitative insights, advanced techniques provide superior sensitivity and specificity for the detection of smaller particles. The article analyses the advantages and limits of these methods, considering factors such as accuracy, cost, sensitivity, and efficiency. It also analyses the basic advantages of artificial intelligence in addressing these limitations. Artificial intelligence's speed, accuracy, and adaptability can enhance microplastic detection and identification, supporting regulatory compliance and food safety monitoring. This comprehensive analysis addresses artificial intelligence's vital role as a future research tool to the rising challenges of microplastic contamination.

## Highlights

• Worldwide occurrence and categorization of microplastics.
• Traditional techniques of microplastic detection.
• Comparative analysis of modern and conventional detection techniques.
• Lacunas and limitations with these techniques.
• The promising role of artificial intelligence in overcoming current challenges.

## 1 Introduction

The presence of microplastic (MPs) contamination is growing at a rapid pace and poses a serious environmental risk. Environmental plastics gradually break down into tiny fibers, fragments, and particles called microplastics (less than 5 mm in size) and are dispersed through the environment (Frias & Nash, 2019). While MPs are elusive and can easily penetrate the human food chain via a variety of sources, such as freshwater, fruits, fish, beverages, etc., they have caused a serious environmental hazard. Recent studies identified MPs in human breast milk, placenta, and blood (Kurniawan et al., 2021). Concerns regarding these MPs are growing, especially due to the excessive use of polymer products in a circular economy. The worldwide presence of MPs underscores the serious threat posed to the environment (Federico et al.). From 1950 to 2015, 6300 million tonnes of plastic waste were generated globally (Geyer et al., 2017). Projections indicate that, between 2015 and 2060, global plastic trash will increase to 270 million tonnes (Xiang et al., 2022). They come from a variety of sources and can be categorized into two main categories primary (used in medical products, toothpaste, cosmetics, and microfibers) and secondary MPs (breakdown of larger plastic items, bags, bottles, fishing nets, degradation by sunlight, and physical abrasion) (Babita, 2023). The most common plastics that became microplastic pollutants were reported to be polypropylene (32 %), polyethylene terephthalate (25 %), polystyrene (22 %), and polyethylene (21 %) (Baruah et al., 2022).

MP particles have been reported to be present ubiquitously in various environments, including freshwater, sediments, soil, and marine ecosystems (Peixoto et al., 2019). Their presence across various ecosystems and within the food chain has raised critical concerns about the potential health risks they may pose to humans (Gasperi et al., 2018; Kadac-Czapska et al., 2023). MPs can contaminate food products through several elementary processes. Firstly, because of their small size, plants frequently absorb MPs and are conveniently consumed by fish, crabs, and terrestrial organisms. Consequently, the food chain enables these MPs to enter the human body (Bradney et al., 2019; Horton et al., 2017). The process and position of an individual's exposure to the particles determine how they affect their health. Secondly, plastic is a usual component used to package food during the processing, shipping, manufacturing, and packing procedures. Plastic particles can come into contact with food intentionally (Kedzierski, Frère, Le Maguer, & Bruzaud, 2020). A single plastic teabag, for illustration, contained 3.1 billion nanoplastics and 11 billion MPs in just one cup of tea while working at the temperature required (Hernandez et al., 2019). Also, MPs have been produced at various steps of food production, like packaging, transportation, processing, and storage (Zhang et al., 2020). The increasing number of ready-to-eat meals, along with examples like MP entering from tea bags, show how packaging affects humans (Zhang et al., 2024). These tiny particles are frequently ingested via the digestive system and harm human health. MPs can serve as carriers for toxic chemicals; they can absorb and accumulate harmful substances, like heavy metals, and other environmental contamination (Zhao et al., 2025). Research has demonstrated that microplastics can cause inflammation and adverse immune responses. Various adverse effects of microplastics with their typical sources are mentioned in Table 2, and the different pathways through which MPs particles enter the human body, their interaction with organs, and sources of microplastic contamination are shown in Fig. 3.

Conventional methods, like optical detection, scanning electron microscopy (SEM), hyperspectral imaging, thermal analytical techniques, and modern techniques like Raman spectroscopy, Fourier transform infrared spectroscopy (FTIR), and pyrolysis-gas chromatography–mass spectrometry (PY-GC–MS) for MP detection (Bitencourt et al., 2020; Nicolai et al., 2022; Xu et al., 2023) have been commonly employed to analyze food samples for plastic pollution (Fig. 4). These techniques have significant limitations, while they have proven effective in certain contexts. These techniques can be time-consuming, making large-scale testing or real-time assessment impossible (Jin et al., 2024). With the limitations of conventional and modern methods, there is a growing need for more improved, adaptable, and cost-effective methods for detecting MPs in food. Artificial Intelligence (AI) describes the capacity of machines to replicate and enhance human qualities, such as gathering data and thought. While AI was initially utilized in computer programs, its potential uses increasingly expanded into an extensive variety of services and products (Zhang et al., 2023). AI-based MP imaging technology, which has been fueled through novel innovation, comprises multiple methods, applications, and developments that are essential to analyzing microplastics (Martin et al., 2018).

**Fig. 1:**
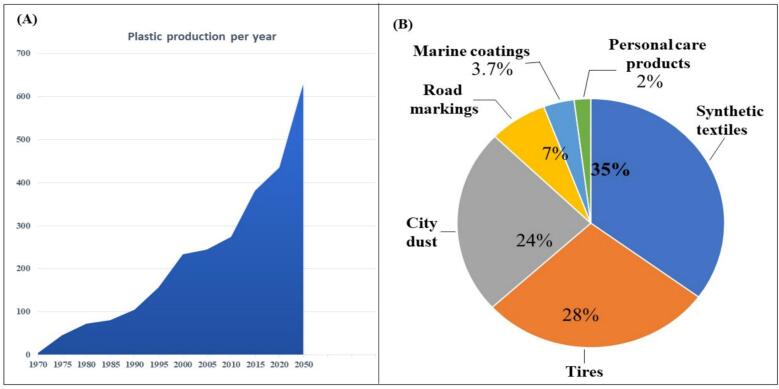
(A) Annual global plastic production trends, measured in million tonnes, demonstrating the rapid increase in plastic production over time. (B) The presence of microplastics in different ecosystems, shown as a percentage distribution across various environmental compartments (source-specific).

These advanced methods have generated significant curiosity due to their several advantages, such as higher efficiency, lower energy consumption, and improvements in conventional imaging methods. They also facilitate examination and provide novel instruments for cognitive and material studies. The application of AI in techniques such as Unmanned Aircraft, robotic systems, and current AI-based algorithms has made it possible to employ greater automation and accurate surveillance methods in the field of microplastic detection and polymer imaging (Bhardwaj et al., 2016). This review examines AI's role in identifying and detecting MPs in food. It includes conventional and modern methods to detect microplastics. Such methods have major inhibitions when it comes to precision and effectiveness. The articles explore the possible advantages of employing AI-based methods, including image recognition and deep learning, to get around these challenges. Based on studies, worldwide production of plastic has grown significantly, and most of the rapid increase occurred around 1990. It indicates a higher need for manufacturing goods for consumers and packaging. Fig. 1.A represents the worldwide production of plastic.

The data graph in Fig. 1. B represents the main source of plastic contamination in the atmosphere, focusing on some of the most important sources according to present studies and environmental surveillance. Synthetic textiles, such as nylon, polyester, and acrylic, were among the biggest contributors to MP pollution, representing 35 % of the overall plastic particles in the atmosphere. As synthetic textiles are processed, small fibers called microfibers get released into the waterways (Acharya et al., 2021). Tires were reported to be the second-largest source, contributing 28 % of MPs (Xiao et al., 2024).

As tires degrade on road surfaces, they provide a mix of plasticizers, rubber, and other additives, which can settle on roads, be washed into stormwater drains, or become airborne as dust, eventually reaching water bodies and soil (Sommer et al., 2018). City dust, which consists of a mix of urban debris, car exhaust, and pollutants from construction, is responsible for 24 % of MPs in ecosystems. This dust often contained particles from degraded plastics such as packaging, textiles, and construction materials (Monira et al., 2022).

Road markings, such as painted lines and symbols, account for 7 % of MP pollution. Such signs that typically consist of thermoplastic substances, paint-based, and derived from plastic, degrade with time and are related to environmental exposure and transportation wear (Burghardt & Pashkevich, 2023). Boats, ships, and marine structures are responsible for 3.7 % of the environment's microplastic contamination. The paint degrades through weathering, friction, and contact with sea water, as it contains substances derived from plastic (Lin et al., 2024). Based on the small particles present in this abrasive therapy, skincare products, including cosmetic products, toothpaste, body washes, and facial scrubs, contributed to 2 % of the MP pollution (Sun et al., 2020).

MPs are frequently detected in food sources like seafood, packaged food, and water, raising health concerns. Current detection techniques have challenges in sensitivity, consistency, and complexity. Scalable, accurate, and standardized techniques, such as AI-assisted tools, are essential to provide effective monitoring and risk assessment of MP contamination.

This review highlights the growing concern regarding MPs contamination in food, driven by the projected rise in global production of plastic, estimated to exceed 2.1 billion tons annually by 2060 (Xiang et al., 2022). This contamination poses serious environmental and human health risks, demonstrating the significance of extensive monitoring and remediation. Table 1 presents data on contamination of microplastics in various food products, sampling origins, microplastic size, shape, types of MPs polymer, and analytical method. The review also explores the primary sources, environmental and health impacts, and classification of microplastics, while providing a comparative analysis of detection methodologies. It examines conventional techniques as well as advanced spectroscopic techniques. This comprehensive analysis provides a valuable system for future research focused on MP detection, risk assessment, and remediation.

**Table 1:** Summary of microplastic contamination in food products. Data includes product types, sampling origins, microplastic sizes, shapes, polymers detected, analytical methods used (e.g., FTIR, Raman spectroscopy), and references.

Product	Sample Origin	Microplastic size (μm)	Shape	Microplastic polymer	Analytical methods	References
Drinking water	Germany	5–1000 μm	Fragments	PA, PE, PET, PP	FTIR spectroscopy SEM- EDX	(Zainuddin & Syuhada, 2020)
Milk	China India United state	5 μm	Fragment, Fibers	PE, PP, PS, PET, PVC	FTIR, Raman spectroscopy	(Badwanache & Dodamani, 2024)
Honey	Switzerland	10–20 μm	Fibers, fragments	PET, Cellulose	Raman, FTIR	(Muhlschlegel et al., 2017)
Sugar	Germany, France, Italy, Spain	10–20 μm	Fragment, Fibers	PE, PP	SEM-EDX, FTIR	(Makhdoumi et al., 2023)
Salt		45–4300 μm		PP, PA, PE	SEM-EDX, FTIR	(Makhdoumi et al., 2023)
Package food	Austria, China, France, Canada	– 0–210 μm 300–450 μm 8.6–52.3 μm	Fibers, fragments, spheres, cylinders - spheres, irregular pieces	PET, PVC, PP, PS	Raman spectroscopy, SEM-EDX	(Kwon et al., 2020)
Fruit and vegetables	Catania	1990–2170 μm	–	–	SEM, FTIR	(Aydın et al., 2023)
Fish	France, Italy	–	–	PET, PC, PTA	Raman spectroscopy	(Di Giacinto et al., 2023)
Soft drinks	–	100 μm	–	PA, ABS, PEA	FTIR	(Altunısık, 2023)
Coffee	–	1000 μm	–	PET, PS, PE	Raman spectroscopy	(Giri, Lamichhane, Khadka, & Devkota, 2024)

## 2 Microplastics: Sources and impact

The widespread occurrence of microplastics has been noticed in various environments, ranging from deep ocean sediments, food, air, to distant soils. This cosmopolitan presence of MP and associated hazards to the ecosystem, food safety, and human health makes it essential to identify the distribution, source, and impacts of microplastic pollution (Tang, Han, Sulaiman, He, & Zhou, 2023; Khalid et al., 2023). The following section is an effort to discuss these factors in detail.

### 2.1 Sources

**Fig. 2:**
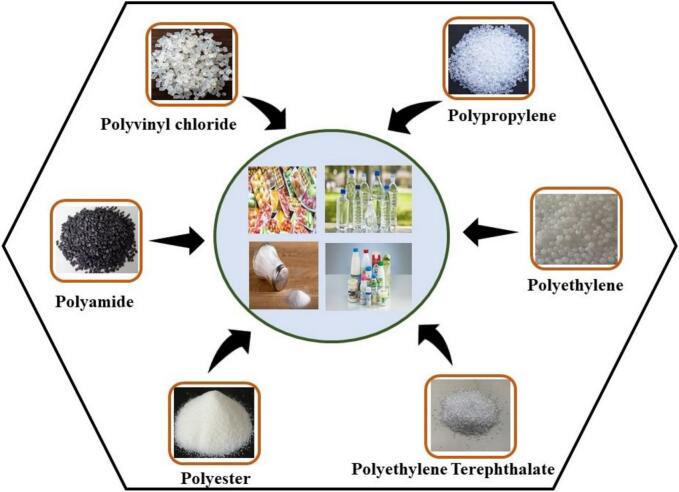
Classification and types of microplastic particles found in food. Categories include fibers, fragments, spheres, and irregular pieces, with size ranges provided where applicable.

MPs in the surroundings primarily originate from two main sources, resulting in different sizes of plastic particles: primary sources and secondary sources (An et al., 2020). Primary sources are manufactured particles detected in products like personal care items. Examples include residues from toothpaste, hair gel, cleansing lotions, and air fresheners (Kadac-Czapska et al., 2024). These particles usually enter the environment due to the discharge of domestic wastewater. Inquiries regarding the presence of MPs in toothpaste have disclosed the presence of polyvinyl chloride (PVC), polypropylene (PP), and polyamide (PA) particles of various sizes from 100 to 399 μm (Anagnosti et al., 2021). Secondary sources were reported as unintentional by-products of plastic degradation, formed using micro (5–25 mm) and mega (>25 mm) plastic debris through various biological, chemical, and physical techniques (Periyasamy & Tehrani-Bagha, 2022). Among these processes, photodegradation has been identified as the most common pathway for the formation of MPs. Along with fragments of plastic debris, including packaging, significant sources of secondary MPs include paint particles shed from many plastic fibers and products released from fishing gear and textiles (Muthu, 2020). Various types of microplastic particles have been reported in foods, and these can be classified based on their shape, size, and origin. These different types of plastic polymers present in foods have been depicted in Fig. 2 with their respective shapes (fibers, fragments, spheres, etc).

**Fig. 3:**
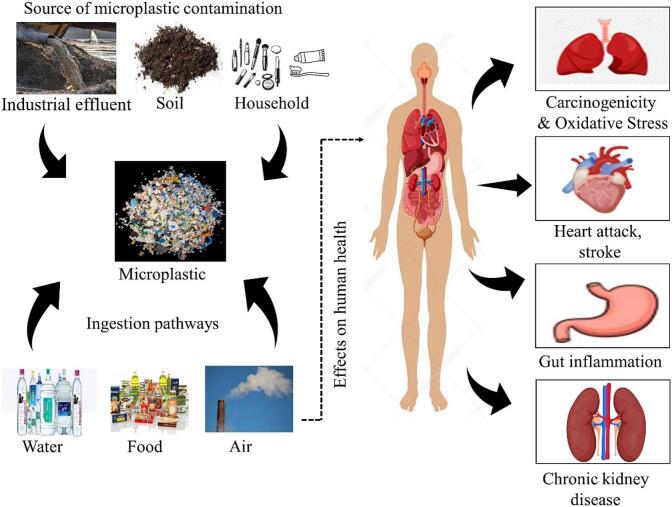
Pathways through which microplastic particles enter the human body, their interaction with organs, and the subsequent health impacts on various physiological systems.

**Fig. 4:**
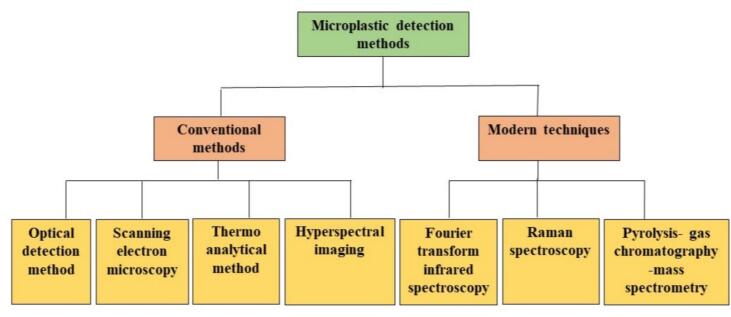
An overview of the methodologies used for detecting and identifying microplastics, including optical detection methods, advanced spectroscopic techniques (e.g., Raman, FTIR), and modern and conventional approaches.

**Fig. 5:**
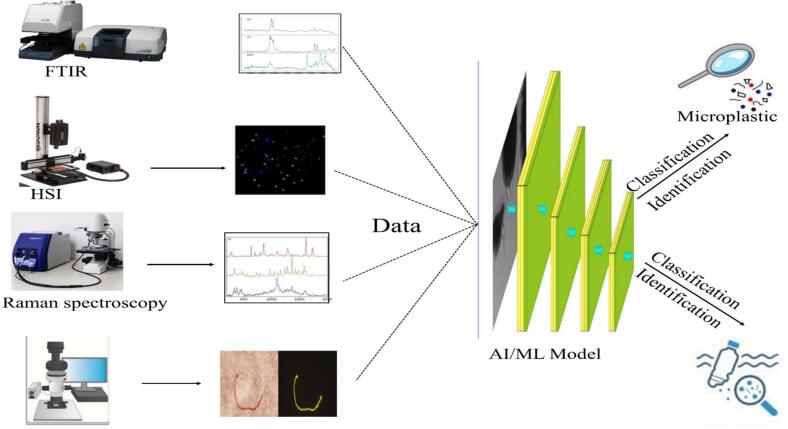
Artificial Intelligence (AI)-Driven Identification and Classification of Microplastics Using Spectroscopic and Microscopic Techniques.

**Fig. 6:**
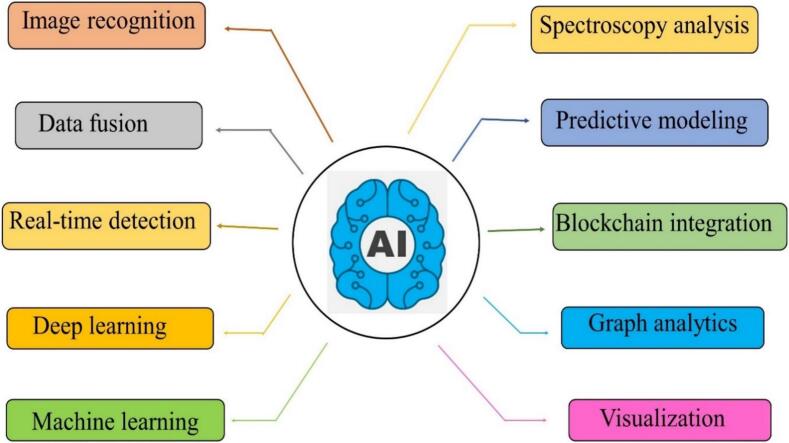
Applications of artificial intelligence in microplastic detection, showcasing various AI-driven methodologies such as machine learning and deep learning for classifying microplastic polymers in environmental and food samples**.**

### 2.2 Pathways of microplastics into the food

MPs can contaminate agricultural soils using plastic mulches, fertilizers containing plastic residues, and irrigation with wastewater (de Carvalho-Souza et al., 2018). Plants can absorb these particles through their roots or have them adhere to their surfaces, entering the food supply. This is especially prevalent for disposable plastics and packaging supplies, which go through pressure or extreme temperatures. Plastic particles may also enter food via plastic surfaces and tools during the process, and they can be absorbed into food during transportation and storage (Kwon et al., 2020). MPs are frequently detected in bottled and tap water due to leaching from plastic bottles and pipes, and may be ingested through food preparation or direct consumption. Airborne MPs may pollute food while processing and consuming, especially in sets that have significant plastic contamination. MPs are mostly created through the innovative breakdown of plastics in the atmosphere, and their tiny size enables them to be simply dispersed in food, soil, containers, and the environment. MPs are ubiquitous in the seas and are taken up by various aquatic organisms, which allows these to get into the food system through various techniques. Seafood, especially shellfish and fish, may accumulate MPs within the body that humans subsequently ingest (Li et al., 2021).

### 2.3 Effects of microplastics on human health

Some research indicated that microplastics can cause cellular damage, which might contribute to long-term health effects like carcinogenesis. MPs can cause acute and chronic toxicity, with a range of harmful effects including reproductive, locomotor, and developmental toxicity, as well as genotoxicity, neurotoxicity, cytotoxicity, and immunotoxicity (Table 2) (Bhagat et al., 2020; Tagorti & Kaya, 2022). Studies have shown that MP exposure triggers oxidative stress, as evidenced by increased antioxidant defenses. Additionally, MPs have been detected within various organisms and on skin, saliva, as well as fecal samples (Ebrahimi et al., 2022).

**Table 2:** Impact of microplastic exposure on human health. Correlates polymer types with specific health effects (e.g., asphyxia, neurotoxicity, cardiovascular damage) and their typical sources.

Polymer type	Health effects	Sources	References
Polypropylene (PP)	Asphyxia	Reusable food containers, single-use face masks, bottle caps, wrap film, and insulation material	(Hwang et al., 2019)
Polyamide (PA)	Injury to the eye	Textile, carpets, sportswear	(Deshoulles et al., 2022)
Polystyrene (PS)	Neurotoxic, respiratory, and skin irritation	Boards of insulation	(Huang et al., 2022)
Polyethylene (PE)	Asphyxia	Wrap film, shopping bags	(Wang et al., 2022)
Polycarbonate (PC)	Hemolysis, hazardous to eyes, causes cardiovascular damage, Skin and respiratory irritation, convulsions, and coma	CDs, DVDs, construction materials, reusable bottles, roofs of greenhouses, glass lenses	(Shi et al., 2021)
Polyvinyl chloride (PVC)	Liver damage, eye hazard, Skin and respiratory irritation are proven carcinogenic	Window frames, shoe soles, pipes	(Qin et al., 2022)
Polyethylene terephthalate (PET)	Kidney damage, irritation to the skin, eyes, and respiratory system	Textile fibers, Bottles,	(Schymanski et al., 2018)

## 3 Detection methods of microplastics

To effectively eliminate diseases, pollutants, or parasitic threats, the foundational step involves the accurate detection and identification of the causative agents. In the context of microplastic pollution, it is imperative to first detect their presence and precisely characterize their type, morphology, and composition before formulating targeted remediation or eradication strategies (Kumar et al., 2024). Over the years, several techniques have been developed to detect and identify MPs in food, ranging from conventional methods such as optical detection, thermo-analytical, and hyperspectral imaging, to SEM, which has been widely used. But these conventional methods often suffer from limitations such as low sensitivity, time-consuming procedures, etc. To overcome these limitations, modern techniques such as FTIR, Raman spectroscopy have been introduced. These methods provide more sensitivity, accuracy, and the ability to identify and classify microplastics at the molecular level (Bhattacharjee & Bhave, 2025). Fig. 4 shows an overview of the techniques used for the detection of microplastics.

### 3.1 Conventional methods

Microplastic detection and identification depend on a variety of conventional methods developed over recent decades. These methods are aimed at examining and classifying microplastics based on their chemical, physical, and optical characteristics (Fernandes et al., 2025).

#### 3.1.1 Optical detection method

The optical detection method is commonly used to identify MPs because of its simplicity and speed. Each microplastic particle was classified depending on its shape, color, and kind, provided it was larger than 500 μm and detectable to the human eye (Renner et al., 2018). These methods employ optical microscopy and other optical tools, frequently combined with fluorescent dyes, to determine and characterize microplastic particles. Due to the relative simplicity of providing application, cost, and rapid outcomes, optical detection methods have seen significant growth in this field. In focusing a beam of laser light over the pattern, it generates an individual spectrum, which is mainly determined by the sample's chemical composition. While becoming easy and readily available, the method cannot be used as a regular detection method, as manual or microscopic count may result in errors and miscalculations. Also, it may prove impossible to count each of the MPs that may include waste (Sridhar et al., 2022).

#### 3.1.2 Scanning electron microscopy (SEM)

SEM is used to examine the morphology and surface structure of MPs. It employs to focuses on the electron source that interacts with the outermost layer of MPs, producing data that show their texture, shape, and surface defects (Ramezani et al., 2024). Various research works have been carried out on applying SEM to MP detection. For illustration, a study on the resolution of MPs in aquatic fish categorized the search pattern into various types, primarily films, foams, fibers, pieces, and micro-pellets (Anderson et al., 2017). Information on chemical composition can also be obtained when equipped with Energy-Dispersive X-ray Spectroscopy (EDS) detectors (Kutralam-Muniasamy et al., 2020). EDS is commonly considered another technique to SEM, but it includes a further detector, enabling the quality and quantitative elemental analysis of the tested sample. Its operating mechanism involves the electricity produced via the cathode of an electron microscope (Azooz et al., 2025). Whenever the primary electron beam impacts the sample's surface, several interactions occur, particularly the emission of X-rays. These X-rays yield data on the chemical composition and spatial distribution, which are characteristic of the sample. This type of microscopy may produce high-resolution topographical images (>0.5 nm) of the sample's surface (Kalaronis et al., 2022). SEM requires extensive sample preparation, experienced employees, substantial equipment, and upkeep expenses. SEM additionally needed an adequate distance to prevent pollution from mechanical and electrical contamination generated by nearby equipment (Crawford et al., 2017).

#### 3.1.3 Thermo-analytical methods

Thermo analytical methods are techniques for analyzing which determine whether a material's chemical and physical characteristics vary with temperature. These approaches are especially successful in identifying and evaluating plastic particles in food (La Nasa et al., 2020). Chemical transformation of MPs may be utilized to determine the various kinds of polymers, along with the organic substances, gases, and chemicals inside the complicated system. This method frequently involves quantitative results at a particular temperature and the absence of oxygen (Yakovenko et al., 2020). These elements are employed alongside gas chromatography and mass spectrometry methods to detect polymers at the molecular scale. In the past few years, this technique has been effectively used on an extensive variety of food products (Fries et al., 2013).

#### 3.1.4 Hyperspectral imaging (HSI)

Hyperspectral imaging shows an unusual method for detecting microplastics. The method employs the spectral examination or image spectroscopy to deal with the variation of reflectivity level throughout image collecting (Shan et al., 2018). The initial objectives of HSI were to study the surface of the Earth. However, it continues to be employed to determine substances for an assortment of purposes (Lu et al., 2020). HSI is commonly utilized in the waste management sector to reduce plastic using polymers. It presents significant evidence for the concept's obvious relation with microplastic research (Zhu et al., 2020). HSI can show physical as well as chemical data and is composed of several spectral bands of light as well as billions of physical components covering the visible-to-thermal spectrum (Huang et al., 2021).

Every technique possesses different advantages, like chemical identification, high resolution, but they also have significant limitations, such as the potential for human error, time-consuming processes, cost effectiveness, etc.

### 3.2 Modern techniques

With microplastic research advancements, more complicated analytical techniques have been developed to overcome the limitations of conventional techniques. Modern techniques aim to increase accuracy, sensitivity, and chemical specificity, providing the detection and identification of MPs particles (Joshi et al., 2024).

#### 3.2.1 Fourier transform infrared spectroscopy (FTIR)

For several years, FTIR has become frequently employed in exploring the distinct characteristics of different compounds. The specimen collected a specific wavelength of infrared energy, leading to errors that are helpful in the identification of molecular features and structures (Sridhar et al., 2022). The FTIR may be characterized by stating that an actual filtration membrane was employed to keep the tiny plastic particles resistant to other particles. The data provided is employed during the subsequent phases within the polymer classification method to determine and verify the maximum quantity of plastic and non-plastic (Li et al., 2018; Nguyen et al., 2019). Also, microplastics have been identified within an assortment of food and drinks, like honey, milk, and beer, employing the FTIR technique (Diaz-Basantes et al., 2020).

#### 3.2.2 Raman spectroscopy

A common method for identifying MPs uses Raman spectroscopy. This technique involves a source of light having a particular wavelength to show the necessary particles through the dispersion process (Imhof et al., 2016). This technique, such as chemical fingerprints, enables the identification of components inside an object (Araujo et al., 2018). This method provided an extensive assessment of food quality in a secure, natural, and rapid manner (Yang et al., 2017; Yaseen et al., 2017). A typical instance of vibrational spectroscopy is the Raman spectroscopy method, which employs a rigid distribution of radiation to generate a vibration spectrum that provides details about the movement of a substance in particles. In this technique, Raman dispersion occurs simply by guiding a beam of light across the substance with an objective lens (Giridhar et al., 2017; Petersen et al., 2021).

#### 3.2.3 Pyrolysis-gas chromatography mass-spectrometry

Hazardous analysis via mass spectroscopy, while the burning of a probable polymer has grown more common (Anuar et al., 2022). The PY-GC–MS technique involves a minimum of sample preparation, and a rapid injection becomes a viable option based on the elemental matrix (Fischer & Scholz-Boottcher, 2017). The ongoing discussion regarding dimension categorization and worldwide uniformity has impeded studies on the movement and transfer of plastic particles. This technique deals with the problem of possible polymer underestimation because it is relevant to any particle size and gives data regarding the amounts of polymers in the samples (Di Fiore et al., 2024).

Although there are conventional and modern methods of identifying microplastics, these frequently face limitations that prohibit accurate, effective, and sustainable detection. AI gives multiple benefits beyond conventional techniques.

Several studies have used **PY-GC–MS** for MP assessment, particularly in agricultural and soil contamination assessments, as seen in the **Tunisia farmland study**, where this method was used alongside **ATR-FTIR** to identify polymer types within soil micro- and macro-aggregates. Similarly, **ATR-FTIR and μ-Raman spectroscopy** have been widely applied in air, soil, and water studies, including airborne MPs research in **Islamabad, Pakistan**, and the **Ross Sea, Antarctica**, where these techniques confirmed polymer composition with high precision (Cincinelli et al., 2017). **SEM-EDX analysis**, as reported in multiple studies, provided insights into morphological degradation and elemental composition, highlighting weathering features like grooves and pits on MP surfaces. Additionally, **NIR-HSI**, as demonstrated in an environmental monitoring study, emerged as a high-throughput method capable of rapidly detecting MP particles with a high degree of accuracy across different polymer types (Faltynkova & Wagner, 2023). The integration of these methods in diverse environmental matrices, including air (PM2.5), soil, seawater, and sediments, underscores their importance in MP research (Pipkin et al., 2021). To enhance the clarity of these findings, Table 3 has been added to provide a systematic comparison of studies, detailing the number of investigations using each method, the specific analytical approaches, and the types of samples examined.

**Table 3:** Studies utilizing microplastic analysis with PY-GC–MS, FTIR, Microscopic imaging, Hyperspectral imaging, and Raman spectroscopy.

Sample type analyzed	Method used	Key findings	References
Freshwater river sediment	PY-GC–MS	>700 particles (polyethylene and polypropylene) per kg of dry sediments were identified	Nel et al., 2020
Agricultural soil	PY-GC–MS	Identified microplastic particle PET, PS (size range200-400 μm)	Chouchene et al., 2022
Marine sediment	PY-GC–MS	High prevalence of Polyethylene and polypropylene has been noticed in marine sediments	Fries et al., 2013
Ocean water	PY-GC–MS	Most detected microplastics were Polystyrene and polyethylene	Pipkin et al., 2021
Marine water	FTIR	Confirmed the presence of various types of microplastic, with a predominant abundance of polypropylene and polyethylene	Cincinelli et al., 2017
Drinking water	Microscopic imaging	Identified microplastic contamination in mussels (PVC and Polypropylene)	Karki et al., 2024
Urban dust, air samples	Raman spectroscopy	Findings revealed a diverse range of microplastics within the dust sample (PP, PE, PA, etc)	Din et al., 2024
Marine ecosystem	Hyperspectral imaging	Identified microplastic four most relevant polymers (PP, PE, PET, and PS)	Faltynkova & Wagner, 2023

## 4 Artificial Intelligence in microplastic detection and identification

AI refers to the limitation of human intelligence by machines that enables them to perform activities such as reasoning, learning, and problem-solving. AI has become an invaluable tool in scientific research because of the rapid growth of machine learning, deep learning, and image recognition. The basic purpose of using AI in MP detection is to overcome the limitations of conventional methods, which are frequently time-consuming, cost-ineffective, and susceptible to human error (Huang & Ullah, 2025). AI is changing the detection and identification of microplastics by offering a more rapid, more accurate, and sustainable alternative to conventional analytical methods. AI can assess microscopic images and spectral information to automatically recognize MP particles based on their size, shape, color, and polymer composition. AI-driven techniques significantly decrease manual effort and human error and allow real-time or large-scale monitoring of microplastic pollution in various environments (Jin et al., 2024). Previous research has shown that artificial intelligence-based technologies can significantly improve detection accuracy and efficiency (Hamza et al., 2022). Fig. 5 shows the path to use AI for the study of data received from spectroscopic and microscopic techniques for the classification and identification of different kinds of microplastics.

AI's ability to analyze enormous quantities of information from multiple sources, like temperature areas, satellite images, and remote sensors, gives it an essential instrument for environmental management (Yuan et al., 2024; Tikle, Jidesh, & Smitha, 2023). AI can effectively detect and identify a wide variety of chemicals, like soil heavy metal pollution and microplastic contamination (Jin et al., 2024). Fig. 6 shows the process of AI in microplastic detection.

### 4.1 Case studies of AI applications

Cowger et al., (2023) studied deep learning methods to determine polymers such as polyethylene and polypropylene via analysis of a dataset of 64,000 Raman bands (Ivleva, 2021). Such AI-driven methods improve traditional error minimization methods and time efficiency (Bayo et al., 2020).

Boyle and Örmeci (2024) explored an adaptable AI system for microplastic identification and detection that employed image capturing and analysis methods. They examined variables like quantity, color, and size, as well as quantities repeated in an environment. The small and easy-to-use technology, driven by a machine learning technique, is suitable for citizen science and scientific study, especially when identifying higher (≥0.5 mm) colored plastic particles (Han et al., 2020). Singh et al., 2023 created an immediate microplastic particle identification technique utilizing portable machine learning and FTIR. This research was to find essential polymers like polyethylene, polystyrene, and polyethylene terephthalate.

Zhang et al. (2022) studied the prospective use of HSI and machine-learning techniques to identify microplastics such as polyamide, polystyrene, and polyethylene. This method detected multiple polymers in environmental samples. Table 4 provides several research findings based on AI microplastic detection and identification.

**Table 4:** Review of AI-powered microplastic detection techniques. The table details AI technologies, methodologies, polymer types identified, and references for studies utilizing machine learning and deep learning for improved detection accuracy**.**

Study	AI Technology	Polymer identified	Methodology	References
AI-Driven FTIR Analysis	Machine Learning	PE, PP, PS, PVC, PET	FTIR spectra were analyzed using Random Forest (RF).	Boyle & Örmeci, 2024
Deep Learning for Image-Based Detection	Deep Learning (CNN)	PE, PET, PA, PS	Microscopic images of particles processed with CNNs.	Cowger et al., 2023
Hyperspectral Imaging with AI	Support Vector Machine	PE, PP, PS, PA, PET	Hyperspectral imaging data was analyzed with SVM.	Zhang et al., 2022
AI in Soil Microplastic Studies	Machine Learning	PP, PET, PVC	Soil samples were analyzed via RF using FTIR data.	Xu et al., 2023
AI for Water Samples	Deep Learning	PE, PP, PS, PET, PVC	Water samples were analyzed using CNN-based pipelines.	Lee et al., 2023
Real-Time Detection with AI	Machine Learning	PE, PET, PP, PS	Real-time analysis using portable FTIR and ML.	Singh et al., 2023

## 5 Applications of artificial intelligence

Machine learning methods may be utilized to detect and categorize plastic particles in food items. Techniques based on deep learning might be developed from huge amounts of images to accurately identify and classify different kinds of microplastic (Zhang et al., 2023). Infrared analysis is frequently carried out to assess the amounts of plastic particles in food products. Machine learning techniques may be developed to identify the spectral characteristics connected with different polymer compounds, enabling automated identification (Weisser et al., 2022). A system driven by AI can monitor food samples in real time using microplastics (Post et al., 2021). AI has been utilized to create predictive models that analyze the probability of microplastic pollution in different kinds of food (Astray et al., 2023). Blockchain integration connects AI-driven detection techniques using digital ledgers to create open and measurable chains of supply. By recording data on MP contamination at each stage of production and distribution, stakeholders can identify sources of contamination and implement targeted interventions (Verma et al., 2022; Yasin, Akkermans, & Van Impe, 2022; Lin et al., 2022). The performance of conventional computer vision techniques is improved via the use of deep learning methods, as is even the use of manual categorizing, which is prone to errors since it is laborious and repetitive. The suggested methodology is suitably quick even when operating on computers without a graphics processing unit (GPU), even though deep learning architectures were better suited to be implemented in computers with GPUs (Lorenzo-Navarro et al., 2021).

Studying the application of AI in microplastic detection is essential for understanding how modern technologies can overcome the limitations of conventional techniques. Methods like image recognition and deep learning simplify the detection and classification of microplastics in images of water, sediments, reducing human errors in differentiating plastics from other particles, and rapidly processing huge quantities of data (Zhou et al., 2024). While machine learning techniques help to analyze characteristics such as size, shape, and spectroscopic patterns essential to categorize MPs' presence and their types (Yang et al., 2024).

## 6 Comparative analysis of artificial intelligence-based modern techniques with conventional techniques

Over the past few years, AI technology has become a vital tool in addressing the major problem of microplastic contamination, offering advanced methods for their analysis, detection, and mitigation (Jin et al., 2024). MP particles that penetrate ecosystems provide serious environmental risks because of their potential and persistent harm to human health, organisms, and diversity (Dekiff et al., 2014). The paper is focused on exploring the role of AI in comparison with conventional and modern techniques (Lin et al., 2022). Conventional methods, such as microscopy and basic spectroscopy, provide high accuracy, direct visual and chemical identification of MPs.

But they are time-consuming and vulnerable to human mistakes. Modern techniques such as Raman spectroscopy and FTIR give specific identification of MPs, which allows polymer-specific detection even in mixed samples. They are limited by their high cost and the requirement for extensive sample preparation. AI-based techniques provide accurate, rapid and scalable analysis. But also come with limitations like requiring a large dataset, advanced computing resources, and challenges with model transparency (Jin et al., 2024).

Several studies have examined AI compared to conventional and modern techniques in different fields. Esteva et al. (2017) studied that deep learning techniques can match a dermatologist to identify skin cancer from images, showing AI's potential as a separate diagnosis tool. Topol (2019) has studied how AI may mitigate human error and enhance diagnostic precision, potentially outperforming human employees. However, it illustrates that AI is more than a supplementary tool for different methods, but it can also be effective alone. This review provides a comparison that highlights the advantages of AI's efficiency, scalability, and sensitivity, demonstrating it as a potential alternative to conventional techniques. Although the study outlines several methods for identifying MPs in food, but more structured analysis is required to assess their practical relevance.

## 7 Challenges and limitations of implementing AI

AI-based microplastic detection in food is challenged by the lack of high-quality, well-annotated datasets that wisely represent the diversity of food matrices and contamination levels. The complexity of food samples, combined with the variability in microplastic structure and composition, provides reliable recognition and classification, often reduces model accuracy. While techniques such as FTIR and Raman spectroscopy provide rich analytical data, their integration into AI frameworks demands substantial technical adaptation and interdisciplinary collaboration. Also, the use of AI systems requires advanced computational resources and specialized abilities, which may limit their access in many research and regulatory contexts.

Though AI has the potential to enhance the performance and accuracy of MP detection, it can't replace basic techniques such as microscopy and spectroscopy, which are required for material identification and characterization. In contrast, AI acts as an additional tool by improving image and spectrum data interpretation, improving specificity, reducing human error, and enabling high-throughput analysis.

## 8 Conclusion

The presence of MP particles in the food chain poses a serious risk to both human health and the environment. As consumers become more conscious of this major problem, there will be a need for more efficient and accurate methods of detection and identification. Conventional methods frequently lack the required capability, awareness, and speed, stressing the requirement for new models. Artificial intelligence may represent cutting-edge techniques for preventing MP pollution. AI employs machine learning and modern techniques to enhance detection efficiency and accuracy, enabling rapid examination of complicated food matrices. This increases identification methods and enables researchers or regulators to measure MP levels, which improves the development of regulations and educational efforts. The difference between conventional and modern techniques highlights the significance of AI-driven strategies for future studies, leading the way for more effective techniques for managing the MP challenge. AI-driven developments in the coming years will significantly enhance the detection, identification, and handling of plastic particles in food, leading to a healthier and safer worldwide food structure, given the reality that currently, there remain challenges to entirely integrating AI for regular food inspection.

## CRediT authorship contribution statement

**Himani Rawat:** Writing – original draft, Validation, Resources, Methodology, Investigation, Formal analysis, Data curation. **Ashish Gaur:** Writing – review & editing, Visualization, Software, Resources, Formal analysis, Data curation. **Narpinder Singh:** Writing – original draft, Validation, Software, Methodology, Investigation, Formal analysis. **Manickam Selvaraj:** Writing – review & editing, Validation, Resources, Formal analysis, Data curation. **Arun Karnwal:** Writing – original draft, Software, Project administration, Investigation, Formal analysis, Conceptualization. **Gaurav Pant:** Writing – original draft, Visualization, Validation, Supervision, Conceptualization. **Tabarak Malik:** Writing – original draft, Resources, Methodology, Formal analysis, Data curation.

## Declaration of competing interest

The authors declare that they have no known competing financial interests or personal relationships that could have appeared to influence the work reported in this paper.

## Data Availability

No data was used for the research described in the article.
